# Symptoms and Their Interpretation

**Published:** 1910-06

**Authors:** 


### Reviews of Books.

---

**Symptoms and their Interpretation.** By JAMES MACKENZIE, M.D., M.R.C.P. Pp. xx, 297. London: Shaw and Sons. 1909.

In the list of writers to whom Dr. Mackenzie in his preface expresses his indebtedness occurs the name of Hilton, and this book on *Symptoms and their Interpretation* might very well be looked upon as an appendix to that author's world-renowned work on *Rest and Pain*. The whole object of the author is "to draw attention to the valuable aid to diagnosis afforded by the careful study of pain, and the nervous phenomena which accompany it," and writing from the stand-point of the general practitioner, he exhorts those engaged in the every-day work of the practice of physic to take up their position as "investigators, and to carefully investigate the reflex symptoms of disease which lie ready to their hands, as it often is on these alone they have to rely for diagnosis and treatment," the more intricate methods of examination of the laboratory or well-equipped hospital often not being available.

The first few chapters are given up to general considerations, such as classification of symptoms of disease, pain, visceral pain, the viscero-motor reflex, etc., after which come chapters on the symptoms of disease of the various organs, a chapter being given to each organ, the stomach, liver, urinary system, etc. In every case valuable suggestions as to diagnosis will be found, special stress being laid on the situation of the pain, and the value to be attached to hyperalgesia of the skin and its subjacent structures in various localities. For instance, in gastric ulcer the author claims to be able to localise the situation of the ulcer; thus if the pain and hyperalgesia be in the upper part of the epigastrium the ulcer is in the cardiac end of the stomach, if in the mid-epigastrium in the middle of the stomach, and if at the lowest part of the epigastrium it is at the pylorus. Again, in gall-stone colic hyperalgesia over the region of the liver may be made out during the attack, or it may persist for some weeks after an attack, and this symptom may be of the greatest use in differentiating an attack of gall-stone colic, attacks which are by no means always easy to diagnose.

In these days of book-making with copious extracts from other works—more especially German—and reports of researches in laboratories, it is quite refreshing to come across an author who gives an account of his own work and his own conclusions written in a breezy and fascinating style that carries the reader along with him. The book is to be cordially recommended to medical readers, to the student as an incentive to him to be accurate in his observations and in reporting cases, and to every medical man engaged in the active practice of his profession, as he will find the interpretation of various symptoms here put before him of the greatest use in his daily work. Dr. Mackenzie is known everywhere in the medical world for the colossal work he has done on disease of the heart, done, be it remembered, whilst he was engaged in the drudgery of a large general practice, and this small book on *Symptoms and their Interpretation*, though of less magnitude than his other works, cannot but add to his reputation.

